# Clinical Management of Implant Prostheses in Patients with Bruxism

**DOI:** 10.1155/2012/369063

**Published:** 2012-06-04

**Authors:** Osamu Komiyama, Frank Lobbezoo, Antoon De Laat, Takashi Iida, Tsuyoshi Kitagawa, Hiroshi Murakami, Takao Kato, Misao Kawara

**Affiliations:** ^1^Department of Oral Function and Rehabilitation, Nihon University School of Dentistry at Matsudo, 2-870-1 Sakaecho-nishi, Matsudo 271-8587, Japan; ^2^Department of Oral Kinesiology, Academic Centre for Dentistry Amsterdam (ACTA), University of Amsterdam and VU University Amsterdam, Gustav Mahlerlaan 3004, 1066 1081 LA Amsterdam, The Netherlands; ^3^Department of Oral Health Sciences KU Leuven and Department of Dentistry, University Hospitals KU Leuven, Kapucijnenvoer 7, 3000 Leuven, Belgium; ^4^Department of Oral Implantology, Nihon University School of Dentistry at Matsudo, 2-870-1 Sakaecho-nishi, Matsudo 271-8587, Japan

## Abstract

There is general agreement that excessive stress to the bone-implant interface may result in implant overload and failure. Early failure of the implant due to excessive loading occurs shortly after uncovering the implant. Excess load on a final restoration after successful implant integration can result in physical failure of the implant structure. Many clinicians believe that overload of dental implants is a risk factor for vertical peri-implant bone loss and/or may be detrimental for the suprastructure in implant prostheses. It has been documented that occlusal parafunction, such as, bruxism (tooth grinding and clenching) affects the outcome of implant prostheses, but there is no evidence for a causal relation between the failures and overload of dental implants. In spite of this lack of evidence, often metal restorations are preferred instead of porcelain for patients in whom bruxism is presumed on the basis of tooth wear. The purpose of this paper is to discuss the importance of the occlusal scheme used in implant restorations for implant longevity and to suggest a clinical approach and occlusal materials for implant prostheses in order to prevent complications related to bruxism.

## 1. Introduction

 The most important factor in implant longevity as a factor for clinically successful implant treatment is the formation of a direct interface between the implant and the bone, without intervening soft tissue, a process called “osseointegration”. Osseointegrated dental implants represent an advance in modern odontology, which has become a great option for the rehabilitation of missing single teeth in partially or totally edentulous patients. Despite the very high success rates [1], complications associated with implant treatment may occur. Early loading failure may affect 2% to 6% of implants, and as many as 15% of restorations fail as a result of this problem [2, 3]. Excess load on a final restoration after successful implant integration can result in failure of the implant itself [4]. Therefore, it is important to clarify the risk factors for failure of implant prostheses in order to further improve the good success rate.

 The consequences of overload of dental implants can be divided into two groups: biological and biomechanical complications [5]. Biological complications can be divided into early failures and late failures [6]. In case of early failures, osseointegration was insufficient: the implant is lost before the first prosthetic loading. Late biological failures are characterized by pathological bone loss after full osseointegration was obtained at an earlier stage [7]. Late biological implant failures are associated with overload. Some insight into bone physiology is needed for a proper understanding of these mechanisms [8–11]. In case of biomechanical complications, one or more components of an implant system fail, for example, fracture of an implant itself, loosening or fracture of connecting screws or abutment screws, loosening or excessive wear of mesostructural components in overdentures, and excessive wear or fracture of suprastructural porcelain or acrylic teeth [12, 13].

 Bruxism is a movement disorder of the masticatory system that is expressed, among others, by tooth grinding and clenching, during sleep as well as during wakefulness [14, 15]. Especially, sleep bruxism is a repetitive sleep movement disorder characterized by rhythmic masticatory muscle activity (RMMA) at a frequency of approximately 1 Hz and by occasional tooth grinding. It is reported that during light sleep, most episodes of sleep bruxism (SB) are observed in relation to brief cardiac and brain reactivations termed “micro-arousals”. RMMA is secondary to a sequence of events in relation to sleep microarousals: as a result of the increase in autonomic sympathetic activity, the heart and brain are activated in the minutes and seconds, respectively, before the onset of activity in the suprahyoid muscles and finally, RMMA occurs in the jaw-closing masseter and temporalis muscles [16]. Since SB is controlled by the central nervous system [17], it may be difficult to prevent the occurrence of bruxism events.

 Clinical trials regarding the influence of bruxism on implant prostheses are scarce. Brägger et al. [18] recognized a causal relation between bruxism and fracture of the suprastructure, but they could not show the relation between bruxism and failure of the implant itself. On the other hand, Engel et al. [19] suggested that bruxism never affected the marginal bone loss of the dental implant. From these studies, it is difficult to conclude that bruxism is a risk factor for dental implants. Since most of the clinical research in dental implants excluded subjects with bruxism, there are only few research data on the influence of bruxism on dental implant outcome, and there is still no scientific evidence for a causal relation between bruxism and implant failure.

In this paper, the relation between occlusion and overload of dental implants is described, and the available evidence for a possible cause-and-effect relationship between bruxism and implant failure is discussed. Further, the possibility of clinical management of implant prostheses using an alteration of occlusal materials in the suprastructure and night guards in patients with bruxism is being presented.

## 2. Occlusal Considerations for Implant Prostheses

 Taylor et al. suggested that since mastication is a side-to-side action that does not lend itself to axial loading of teeth or implants in the jaws, the damaging effects of bruxism are created through lateral friction between the occlusal surfaces of maxilla and mandible [20]. The attachment of natural teeth through periodontal ligaments and osseointegrated implants with a rigid bone contact in the jaw presents a significantly different environment, and this needs consideration. A natural tooth can be intruded about 50 *μ*m by a light force (20 N) compared to only 2 *μ*m for an osseointegrated implant [21]. In an animal study, Miyata et al. investigated the relationship between occlusal overload and peri-implant tissue and suggested that peri-implant bone resorption occurred under occlusal overload [22–24]. On the other hand, Heitz-Mayfield et al. demonstrated that a period of 8 months of excessive occlusal load on titanium implants did not result in loss of osseointegration or marginal bone loss when compared with nonloaded implants in animal study [25]. However, much of oral and masticatory function seems to be similar in natural and implant-supported dentitions [26–28]. The periodontal ligament is lost after tooth extraction, but most of its functional role as related to occlusion and mastication seems to be taken over by other mechanisms, such as, muscle spindles, mechanoreceptors in the temporomandibular joints [29]. Since successful long-term results of implant prostheses have been reported repeatedly, it may be concluded that the variety of methods related to occlusal morphology used in fixed prosthodontics on natural teeth are equally acceptable for rehabilitation on dental implants. The simple principles described for conventional prosthodontics may therefore be followed also for implant prostheses [21]. A literature review concluded that the occlusal scheme for an implant prosthesis should be designed to decrease cuspal interferences, centralize forces along the long axis, and minimize lateral forces; that is, it should be like that of a similar prosthesis on a natural dentition [30].

 Since the occlusal perception level is higher for implant prostheses than for natural teeth, complaints of implant patients should be carefully considered when checking their occlusion. It is established that the lack of periodontal receptors leads to impaired fine motor control of the mandible in implant patients [31]. However, early studies concluded that the functional clinical capacity of patients with implant prostheses was almost equal to or approaching that of dentate subjects [32]. A study showing that the tactile sensibility of single-tooth implants opposing natural teeth was similar to that of pairs of opposing natural teeth led to the conclusion that the implants can be integrated in the stomatognathic control circuit [33].

## 3. Bruxism as Occlusal Risk Factors

 In 1996, Lavigne et al. [34] proposed sleep bruxism research diagnostic criteria (SB-RDC) for polysomnographic recording, as follows: (1) a history of frequent tooth grinding occurring at least 3 nights per week for the preceding 6 months, as confirmed by a sleep partner; (2) clinical presence of tooth wear; (3) masseter muscle hypertrophy; (4) report of jaw muscle fatigue or tenderness in the morning. Bruxism is frequently considered an aetiological factor for temporomandibular disorders (TMDs), tooth wear (e.g., attrition), loss of periodontal support, and failure of dental restorations, although conflicting evidence for many of these purported aetiological relationships can be found in the literature [35–40]. Bruxism has also been suggested to cause excessive (occlusal) load of dental implants and their suprastructures, ultimately resulting in bone loss around the implants or even in implant failure. Therefore, bruxism is often considered a cause of concern or even a contraindication for implant treatment. In addition, many researchers use bruxism as an exclusion criterion for the selection of their participants in clinical studies concerning treatment modalities with dental implants [5].

 Bruxism, other oral parafunctions, fractures of natural teeth resulting from occlusal forces, and lateral occlusal contacts on the implant prostheses were listed as important risk factors for dental implants and their suprastructures [41]. In a study of 379 patients who had used implant prostheses for many years, occlusal wear had no statistically significant impact on vertical peri-implant bone loss [19]. It was presupposed that occlusal wear was closely related to bruxism, and thus bruxism did not seem to be a risk factor for the examined variables. Tooth wear does not represent the actual/current bruxism status. It must be emphasized, of course, that bruxism is not the only cause of tooth wear and in fact is not a major factor [42]. A review of literature on dental implants in patients with bruxing habits concluded that, so far, studies on bruxism and implant failure do not yield consistent results [43]. However, a careful approach was recommended, although it was admitted that these recommendations were “experience based,” not evidence based [21].

 As many clinicians still have the impression that there is some relevance in these risk factors, it may be prudent to exercise caution, perform careful clinical control, and acknowledge the need for occlusal adjustments of the suprastructure in all implant patients [21]. When alarm signals are found, for example, repeated loosening or fracture of abutment screws and fracture of veneering material, a careful analysis of potential reasons for these signals should follow with the aim to modify the situation and reduce excessive risks [41].

## 4. Occlusal Material for the Suprastructure in Implant Prostheses

 In the past decades, for implant prostheses, it was strongly recommended to use a shock-absorbing material, such as, acrylic resin on top of the superstructure, in order to protect the implant-bone interface [21]. Based on biomechanical analyses, acrylic resin denture teeth were therefore predominantly used during the initial years of dental implant use [44]. However, biomechanical calculations do not always stand the test in the clinic. In a clinical study on five subjects using fixed prostheses with either acrylic resin or porcelain occlusal surfaces, masticatory forces were recorded while the subjects chewed various foods. No differences related to tooth material could be detected in the load rates [45]. In a study covering 6 years, the use of porcelain instead of composite resin as occlusal material had no influence on the marginal bone height around the implants [46]. These findings can be interpreted as a support for the use of porcelain as occlusal material because no serious biological consequences of the hard material were reported. Furthermore, the most common complications of implant prostheses have been related to fractures of the acrylic resin of the prostheses [47]. Wear of acrylic occlusal surfaces increased substantially with time, according to a 15-year followup of fixed implant-supported prostheses in the edentulous maxilla [48].

**Figure 1:**
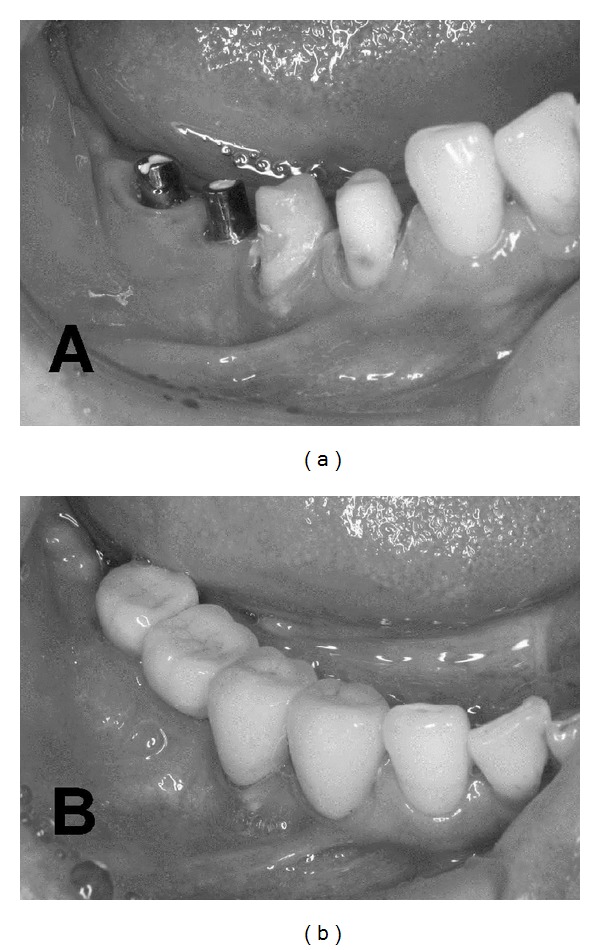
Porcelain has become the primary occlusal material for single-tooth and partial fixed implant restorations.

**Figure 2:**
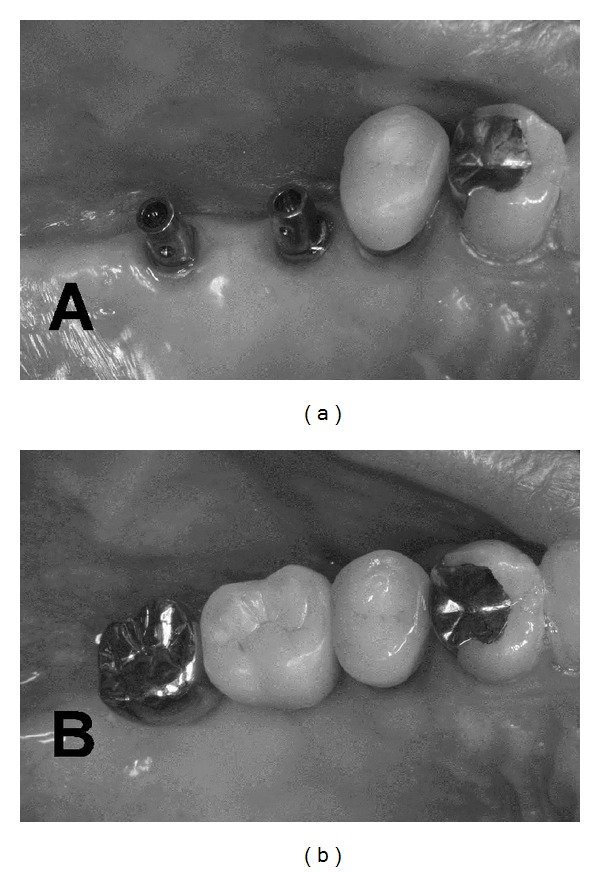
Although there is no evidence regarding the preferred restorative materials in implant prosthesis for the patients with bruxism, some clinicians applied non porcelain but metal restorations to protect the implant prostheses in patients with bruxism, especially for second molar teeth in the maxilla (since there is no esthetic problem).

 In current clinical practice, porcelain has become the primary occlusal material for single-tooth and partial fixed implant prostheses (Figure 1). It is generally agreed that ceramic occlusal surfaces provide superior esthetics and wear resistance [21]. Regarding full-arch fixed prostheses on implants, metal ceramic prostheses are sometimes presented in clinical reports, but in many centers acrylic resin teeth continue to be the material of choice. In removable types of implant prostheses, for example, overdentures, polymer teeth are the most common ones [49]. Although there is no evidence regarding the preferred restorative materials in implant prosthesis for patients with bruxism, some clinicians prefer metal restorations and not porcelain to protect the implant prostheses in patients with bruxism, especially for second molar teeth in the maxilla (Figure 2). Evidently, more clinical trials are needed to provide evidence for these recommendations. More recently, framework or crowns in zirconia was also developed in this field. However, in a clinical trial on fractured dental zirconia implants, Gahlert et al. reported that “the patient with the fracture of the 4 mm diameter zirconia implant was adversely affected by strong bruxism” [50]. Recently, some investigators demonstrated zirconia as a new dental implant material [51–53]. The development of new dental implant material might change the relationship between fractured dental implants and bruxism.

## 5. Night Guard and Pharmacological Approach for Bruxism

**Figure 3:**
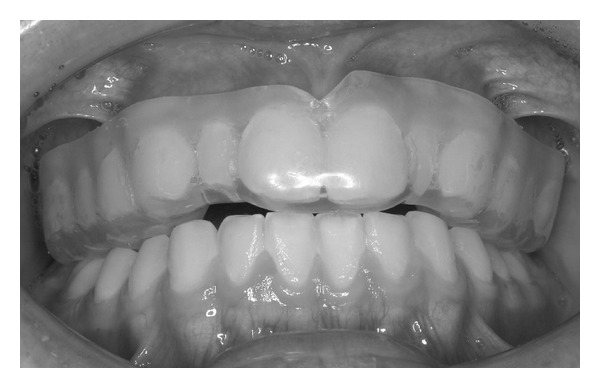
A night guard may be one of the possible solutions to prevent overloading of the suprastructure of implant prostheses. Soft material also could be used for night guards.

A night guard fabricated for the maxillary teeth can be a useful tool to evaluate the influence of the occlusion scheme and its relationship to nocturnal bruxism [4, 54]. Occlusal schemes and designs of fixed and removable implant prostheses must satisfy the requirements for an innocuous vertical loading of dental implants. Parafunctional habits (clenching or grinding) can transmit forces to the supporting bone that may result in destructive lateral stresses and overloading. The consequences of nocturnal parafunctional habits may be prevented by acrylic resin night guards [55]. A hard stabilization splint for nightly use (night guard) contributes to optimally distributing and vertically redirecting forces that go with nocturnal teeth grinding and clenching [5]. A night guard that promotes even occlusal contacts around the arch in centric-related occlusion can be helpful to prevent fractures of implant prostheses. This device may be fabricated with 0.5- to 1-mm colored acrylic resin on the occlusal surface. If the patient wears this device for 1 month, the consequences or intensity of the bruxism habit may be directly observed. If the colored acrylic is not worn through, the parafunction was not excessive [4]. As examples, when partial implant prosthesis is present in the maxilla, the night guard is hollowed out at the implant sites so no occlusal force is transmitted to the implant prostheses. When the partial restoration is in the mandible, the occluding surface of the guard is relieved over the implant prostheses so no occlusal force is transmitted to the implants. A soft material may also be placed around the crowns for stress relief and to decrease the impact force on the crowns (Figure 3). In this field, future clinical trials on possible new materials should be planned to investigate the protection of implant prostheses from bruxism.

On the other hand, some investigators proposed a pharmacological approach for bruxism patients with implant prostheses [56, 57], especially in cases where oral implants failed as a probable consequence of severe, polysomnographically confirmed sleep bruxism. As the patient had the wish to be reimplanted after this failure, the operators decided to try diminishing the frequency of bruxism and duration first. The selected management strategies, the administration of low doses of the dopamine D1/D2 receptor agonist pergolide finally resulted in a substantial and lasting reduction in the bruxism outcome measures under study.

## 6. Conclusion

 Only little research has focused upon the clinical approach to protect implant prostheses from bruxism. The presented results do not reflect a high level of scientific evidence and may need modification when new research results or new dental implant materials appear. It is a fact that clinicians feel that the overload caused by bruxism may result in failure of implant supported prostheses. Following the recent developments with the introduction of immediate or early loading, the clinical management of bruxism will become an important subject for implant prostheses. The lack of well-designed clinical trials regarding the consequence of bruxism on implant prostheses poses a serious problem. At present, expert opinion and cautionary approaches are still considered the best available sources for suggesting good practice indicators. There is an urgent need for those actively engaged in clinical research centers and university research institutes to provide evidence on whether the subjective feeling of clinicians regarding the approach of bruxism in implant patients is correct or not.
